# Studies on acceptance, evaluation and impact of the Cologne program “Research and Medical Studies”

**DOI:** 10.3205/zma001298

**Published:** 2020-02-17

**Authors:** Sören Moritz, Abdul Halawi, Charlotte Proksch, Jan-Michael Werner, Mats Paulsson, Markus Rothschild, Christoph Stosch

**Affiliations:** 1 University of Cologne, Medical Faculty, Vice Dean's Office for Research, Cologne, Germany; 2 University of Cologne, Medical Faculty, Vice Dean's Office for Teaching and Studies, Cologne, Germany; 3 University of Cologne, Medical Faculty, Medical Student Council, Cologne, Germany

**Keywords:** research, medical studies, research track, NKLM

## Abstract

**Introduction:** The curricular implementation of events (or programs) for science-related training in human medicine has been on the agenda of the medical faculties since the publication of the Federal-State Working Group [1]. The Medical Faculty of the University of Cologne developed and established a systematic, longitudinal science curriculum together with the start of the model curriculum in human medicine in 2003. Here, we investigate the questions of whether the described (para-) curricular elements are accepted by students and lecturers and how they are evaluated, especially by students. In addition, we investigate whether selected parameters can be used to demonstrate changes in the students' scientific activities.

**Project description: **The program “Research and Medical Studies” (RaMS) consists of several components: these elements of the mandatory curricular (Scientific Projects, SP) and optional components (Research in Medical Studies (RiMS), Research Track (RT), Research Fair Cologne (RFC)) are described here. Results were recorded at various levels:

1. Likert Scale evaluation of the event’s elements were collected as satisfaction parameters from the students
2. Process data on participation in the voluntary events were collected and evaluated as absolute and relational figures (WS 12/13-SS 17).
3. Data on the outcome of the RaMS program were collected: Type of scientific projects in the academic years 2011/12-2014/15), number and type of available projects offered at the RFC (in the years 2011-18) and number of student research funding applications in a comparison of the periods 2010-13 vs. 2014-17).

**Results: **The students’ acceptance of mandatory and paracurricular courses of the RaMS program is pleasingly high, which is not surprising, at least in the case of the voluntary courses. The participation of students in RiMS, RT and RFC is satisfactory for voluntary courses. In the case of the RT, with certified participation of approximately 47% of all registrations (corresponding to 10% of the total cohort), this is comparable to similar programs. It can be shown that the number of experimental science projects has more than doubled over time in parallel with the development of RaMS. The average number of provided projects according to the RFC is 42 (which corresponds to a placement rate of approx. 1:4). The number of successful student applications for a research support grant during the period the measures were implemented has doubled.

**Discussion and conclusion: **The RaMS program shows a route for the implementation of the SP required by the next licensing regulations in medical education, which was initially supported and expanded solitarily, later by further elements (RiMS), also in the sense of a science-based career development (RT, RFC). The student acceptance and the measured success, in the form of successful participation in the Research Track, increased choice of experimental projects, significant increase of submitted as well as approved research grants and the high project placement rate of the Research Fair, encourage the further development of the program, which is indicated in the conclusion.

## Zusammenfassung

**Einleitung: **Die curriculare Implementierung von Veranstaltungen (bzw. Programmen) zur wissenschaftsbezogenen Ausbildung in der Humanmedizin steht spätestens seit der Publikation der Bund-Länder-Arbeitsgruppe [1] auf der Agenda der Medizinischen Fakultäten. Die Medizinische Fakultät der Universität zu Köln hat mit Beginn des Modellstudiengangs Humanmedizin 2003 ein systematisches, longitudinales Wissenschaftscurriculum entwickelt und etabliert. Hier untersuchen wir die Fragen, ob die beschrieben (para-) curricularen Elemente von den Studierenden und Lehrenden angenommen und wie diese insbesondere von den Studierenden bewertet werden. Zudem wird untersucht, ob sich anhand ausgewählter Parameter eine Änderung der wissenschaftlichen Aktivitäten der Studierenden beschreiben lässt.

**Projektbeschreibung: **Das Programm „Forschung und Medizinstudium“ (FuM) besteht aus mehreren Komponenten: Diese Elemente der pflichtcurricularen (Wissenschaftliche Projekte, WP) und additiven Anteile (Forschung im Medizinstudium (FiM), Research Track (RT), Forschungsbörse Medizin Köln (FBK)) werden beschrieben. Ergebnissicherungen wurden auf verschiedenen Ebenen durchgeführt:

1. Likert-skalierte studentische Veranstaltungskritiken zu den Elementen wurden als Zufriedenheitsparameter erhoben.
2. Prozessdaten zur Teilnahme an den freiwilligen Veranstaltungen wurden erhoben und als absolute sowie als relationale Zahlen ausgewertet (WS 12/13-SS 17).
3. Daten zum Outcome des Programms FuM wurden erhoben: Art der Wissenschaftlichen Projekte den Studienjahren 2011/12-2014 /15), Anzahl und Art der vermittelten Projekte auf der FBK (in den Jahren 2011-18) und Anzahl studentischer Forschungsförderungsanträge im Vergleich der Zeiträume 2010-13 vs. 2014-17).

**Ergebnisse: **Die Akzeptanz der Studierenden von pflicht- sowie paracurricularen Veranstaltungen des Programms FuM ist erfreulich hoch, was zumindest bei den freiwilligen Veranstaltungen nicht verwundert. Die Teilnahme der Studierenden an FiM, RT und FBK ist für freiwillige Veranstaltungen zufriedenstellend, im Fall des RT bei einer zertifizierten Teilnahme von ca. 47% aller Anmeldungen (dies entspricht 10% der Gesamtkohorte) vergleichbar zu ähnlichen Programmen. Es kann gezeigt werden, dass sich im zeitlichen Verlauf parallel zur Entwicklung von FuM die Anzahl der experimentellen Wissenschaftlichen Projekte mehr als verdoppelt hat. Die Anzahl der vermittelten Projekte nach der FBK liegt durchschnittlich bei 42 (was einer Vermittlungsquote von ca. 1:4 entspricht). Die Zahl der erfolgreichen studentischen Anträge auf ein Forschungsunterstützungsstipendium hat sich im Zeitraum der Implementation der Maßnahmen verdoppelt.

**Diskussion und Fazit: **Das Programm FuM zeigt einen Weg für die Implementierung der mit der nächsten Approbationsordnung geforderten WP im Medizinstudium auf, welches anfangs solitär, später durch weitere Elemente (FiM), auch im Sinne einer wissenschaftsbasierten Karriereentwicklung (RT, FBK), unterstützt und ausgebaut wurde. Die studentische Akzeptanz und der gemessene Erfolg, in Form von erfolgreicher Teilnahme am Research Track, vermehrter Wahl experimenteller Projekte, signifikanter Erhöhung von beantragten sowie bewilligten Forschungsstipendien und der hohen Projektvermittlungsquote der Forschungsbörse, ermuntern zur Weiterentwicklung des Programms, welche abschließend angedeutet wird.

## Introduction

The teaching of scientific competence in medical studies is the subject of an intensive, current academic debate [2], [3], [4], [5]. The Federal-State Working Group has released the “Master Plan for Medical Studies 2020” [1], the implementation of which is likely to establish the teaching of scientific skills as an examination requirement in human medical education. The Flexner Report [6], published more than 100 years ago, already described and criticized the profit-oriented and little scientific education of future medical doctors in America at that time. In Flexner's view, formal-analytical critical thinking, which is the basis of all scientific activity, should be the foundation of medical education and also of all medical practice.

The German National Competence-Based Learning Objectives for Medical Education (NKLM) [http://www.nklm.de], adopted by the Medical Faculty Association (MFT) in 2015, following the CanMEDs model [7], contains and requires the role of a “scholar”, science-oriented teaching as well as independent work of a research question for every student of human medicine. Compared with other – divergent – international frameworks for scientific studies in medicine, the role of a scholar in the NKLM thus defines a comparatively stronger methodological orientation [8], [9] as crucial. Thus, the catalogue of learning objectives was accused of being too practice- and school-oriented during its development [10]. In contrast, the German Science Council views the scientifically oriented competencies in the NKLM as a way of firmly integrating the explicit, systematic teaching of scientific competencies in the education of human medicine [11]. Furthermore, the learning goal structure of the NKLM offers the possibility of relatively free design by the faculties and therefore the possibility of creating a “modern” curriculum in every sense, as described by Stern and Papadakis [12]. 

The German Science Council has published “Recommendations for the Further Development of Medical Education”, which, among other things, requires the strengthening of the scientific competence of students [11]. For the latter, “the obligatory acquisition of scientific competence during studies (...) is a necessary prerequisite for the responsible practice of medicine”. It has been shown internationally that scientific programs in medical studies can have a positive influence on subsequent research-oriented career development [13], [14], [15].

Based on these recommendations, the question arises for the medical faculties, how it is possible to establish curricular and paracurricular courses in order to integrate the teaching of scientific competences into the already time-consuming medical studies. 

This thesis aims to answer two research questions in the context of local (para-) curricular implementation:

1. are the described (para-) curricular elements well accepted by students and lecturers and how are they evaluated by students in particular?
2. is it possible to describe a qualitative change in the scientific activities of students on the basis of the selected parameters (types of scientific projects, number of applications in the “Köln Fortune” scholarship program)?

## Project description

**Figure 1:**
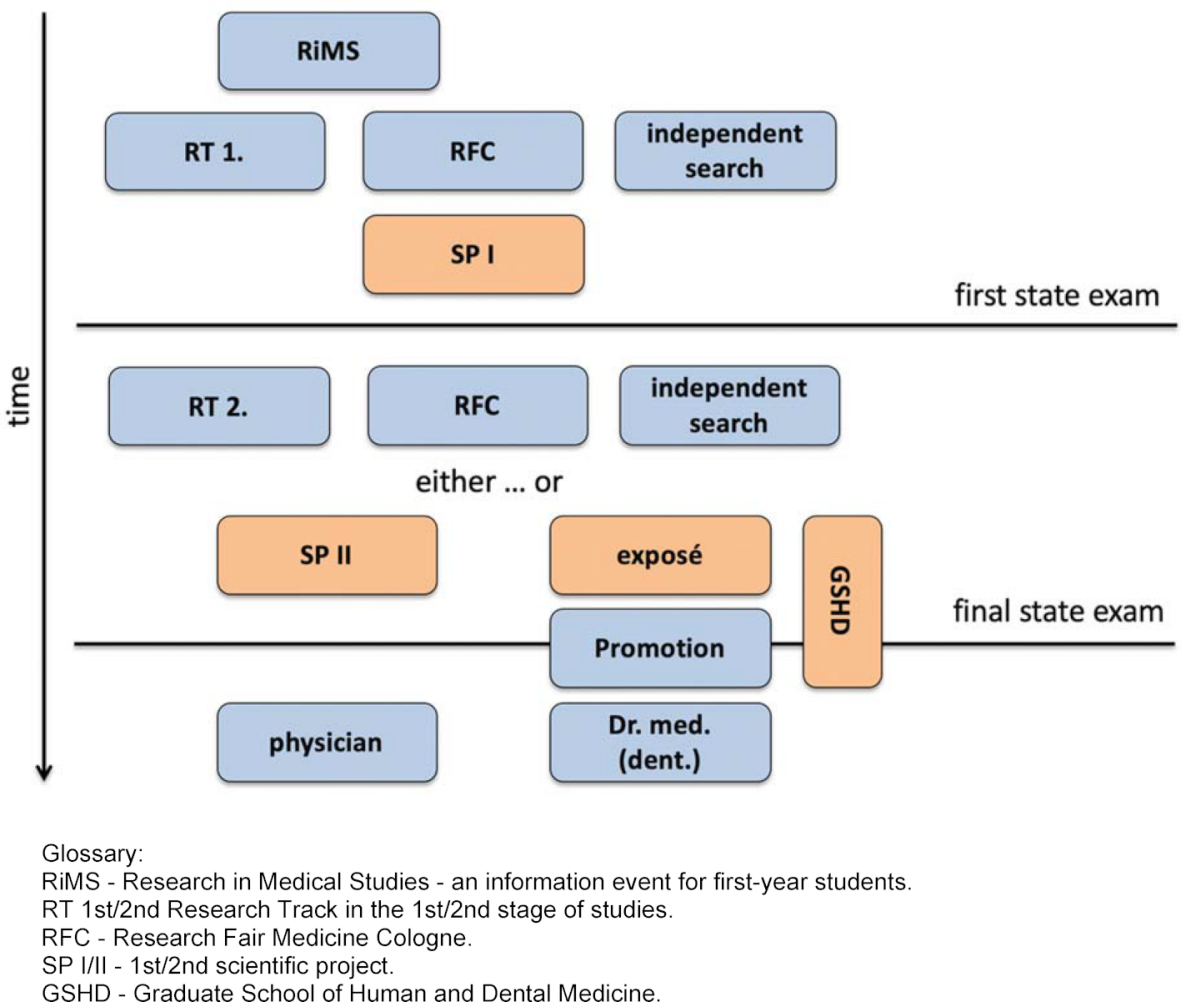
Overview of the science-oriented paracurricular and curricular elements of "Research and Medical Studies" at the Faculty of Medicine of the University of Cologne. Mandatory elements are marked in orange. The paracurricular offerings serve as an introduction to medical research or scientific work and provide an overview of the research landscape. A student can find his or her scientific project via the Research Track, via the Research Fair or via an independent search. This structure continues accordingly in the 2^nd^ study stage. The project outline of the planned doctoral project can be accredited as a Scientific Project II.

Mainly curricular elements were designed, implemented and evaluated as a methodological basis. The elements (for an overview see table 1 (Tab. 1) and figure 1 (Fig. 1)) and the associated evaluation methods are described below. With the exception of the “Scientific Projects” (see below), all courses are open to human and dental medicine students.

### Informative Event – Research in Medical Studies (RiMS)

The informative event RiMS is offered to students in the first semester. Students are given an overview of the research landscape in Cologne, information about the Cologne scientific curriculum and the paths to science by present scientists or researching physicians are shown. 

After this introductory part, students visit research laboratories to gain a first insight into experimental, biomedical fundamental research. In order to evaluate the course, an evaluation sheet with a 6-level Likert scale (liked it – disliked it) was used to assess the individual parts of the course, the overall evaluation of the course by the students and additional characteristics (gender, semester, reason for attendance) (see attachment 1 ). The data was collected continuously for the courses from the winter semester 2012/13 to the summer semester 2018.

#### Research Track (RT)

**Figure 2:**
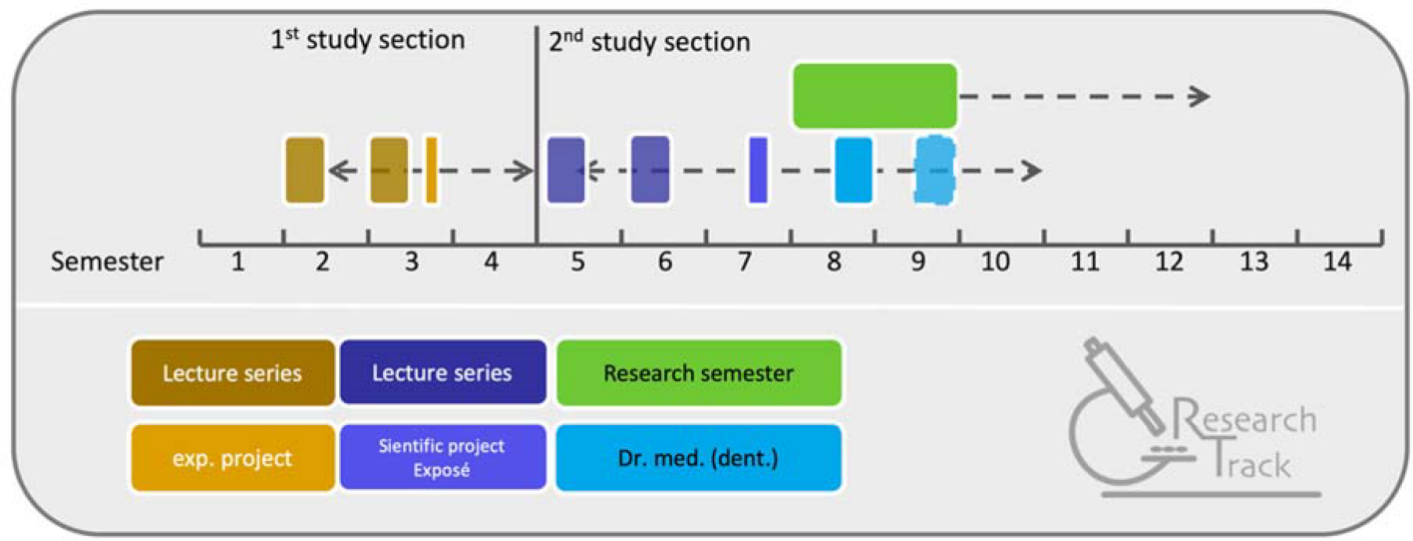
Curriculum of the Research Track (RT). The RT consists in both study stages of a lecture series, which is offered over two semesters. The RT in the 1^st^ study stage includes a mandatory experimental practical internship, which can be accredited as Scientific Project I. In the 2^nd^ stage of studies, the RT should lead to a medical doctoral thesis, whereby the project outline for the doctoral thesis can be accredited as Scientific Project II. In both stages, the students will be actively motivated to take a research semester off for their doctoral thesis and will be informed about the existing funding possibilities within the framework of Köln Fortune and other scholarship providers.

The RT is an optional program that is intended to give students interested in research the opportunity to carry out experimental research projects at an early stage (for a summary see figure 2 (Fig. 2)). 

The RT program in the first stage of study comprises two lecture series held in English in which scientists from the Faculty of Medicine present their research (mainly from fundamental science areas). The core learning objective is to understand the scientific method of developing hypotheses based on a research question and to test these hypotheses experimentally. Practical teaching elements include visits to research laboratories, the presentation and explanation of large-scale research equipment and techniques, and visits to research facilities of cooperating institutions such as the “German Aerospace Center” or the “German Sport University Cologne”. RT participants must complete a final experimental internship of at least 60 hours (this can be accredited as Scientific Project I). The successful participation in the RT will be certified. 

The continuation of the RT In the second stage of study is offered as a “lunch seminar”. Here, the main focus is finding a suitable doctoral project, not obtaining a second certificate. The time course of the RT curriculum is shown in figure 2 (Fig. 2).

In both parts of the RT, a student evaluation was carried out after each course, in which student satisfaction was assessed on a 6-level Likert scale (liked it – disliked it). At the end of the semester, an evaluation sheet was distributed, on which items were assessed according to the time of the event (for 1^st^ stage of studies Wednesdays 6-8 p.m.; for 2^nd^ stage of studies lunch break 1-2 p.m.), the agreement to English-language lectures and other items (see attachment 2 ) and the overall assessment of the event by the participants. The proportion of successful RT participants was expressed as a percentage of the number of students who completed the RT completely (i.e. 20 courses were attended and the experimental internship completed) in relation to the number of students who registered for the RT by e-mail after the first lecture of the respective semester.

#### Research Fair Cologne (RFC)

The RFC is a joint project of the students of the Medical Student Council and the Dean of Studies and Research of the Medical Faculty and has been offered every summer semester since 2013. The DoktaMed of the LMU Munich [16] served as orientation and model for this event and is organized analogous to a scientific poster presentation. The aim of the Research Fair is on the one hand to provide an overview of the currently active research groups of the faculty and on the other hand to enable a direct encounter between students and scientists in order to initiate scientific projects and doctoral theses. In order to evaluate the event, an evaluation sheet with a 5-level Likert scale was developed by the Medical Student Council (liked it – disliked it). This sheet was used to assess the overall evaluation of the event by the students and to ask for additional characteristics (gender, semester, reason for attendance, project found?). The data collection took place between winter semester 2012/13 and summer semester 2018.

In order to measure the success of the project placement by the Research Fair, an outcome evaluation was carried out 6 weeks after the Research Fair, during which the participating working groups were interviewed by e-mail and telephone. The following parameters were recorded: Number of project placements, type of projects and whether a publication is planned. For the sake of simplicity, the project types were divided into the following categories: experimental, clinical-statistical and literature work.

#### Scientific Projects I and II (SP I and II)

SP are an integral part of the Cologne model study program and are equivalent to the electives required in the ÄApprO [17]. In each study stage, each student has to carry out an own scientific project of about 60-160 hours (according to the study regulations). The projects range from humanities and social science topics to clinically relevant questions and basic experimental research. Literature research on medical-scientific questions, clinical-statistical projects, qualitative analyses and, of course, experimental internships can be carried out [18]. The proof of performance is an internship report in the style of a scientific publication. In order to analyze the types of scientific projects, the evaluation sheets in the student files of the examination office from the academic years 2011/12 to 2014/15 were evaluated and assigned to the following categories according to the title of the projects: Experimental projects, clinical-statistical projects, literature work, non-assignable projects, scientific work of other origins acknowledged as projects (acknowledged projects). The acceptance of the SP is reflected in the evaluation results of the students’ satisfaction with the idea of projects as a whole as well as with the projects themselves (pooled data from 4 semesters between 2008 and 2011 (winter semesters 2008/09 and 2011/12 as well as the summer semesters 2008 and 2009)).

Within the framework of the faculty's internal support program “Köln Fortune”, every student has the opportunity to apply for a scholarship for his or her work on research projects. Although information about the possibility of student support was already provided, targeted information for interested students was only started with the introduction of RiMS, RT and RFC. The projects will be evaluated by the “Köln Fortune Research Advisory Council” according to their scientific quality and, if appropriate, approved. The number of approved grants was taken from the respective annual reports of “Köln Fortune”.

## Results

In order to give an overview of the different measures, the temporal course of the scientifically-oriented paracurricular and curricular information and teaching programs is shown in figure 1 (Fig. 1). In addition, table 1 (Tab. 1) contains a short description of each individual program for better comprehensibility. The total number of students per semester is 189 in the pre-clinical stage and between 165 and 175 in the clinical stage. Although the courses are open to students of human medicine and dentistry, we have observed that they are almost exclusively attended by students of human medicine. Therefore we are focusing on these students in this paper. 

### Research in medical studies (RiMS)

**Figure 3:**
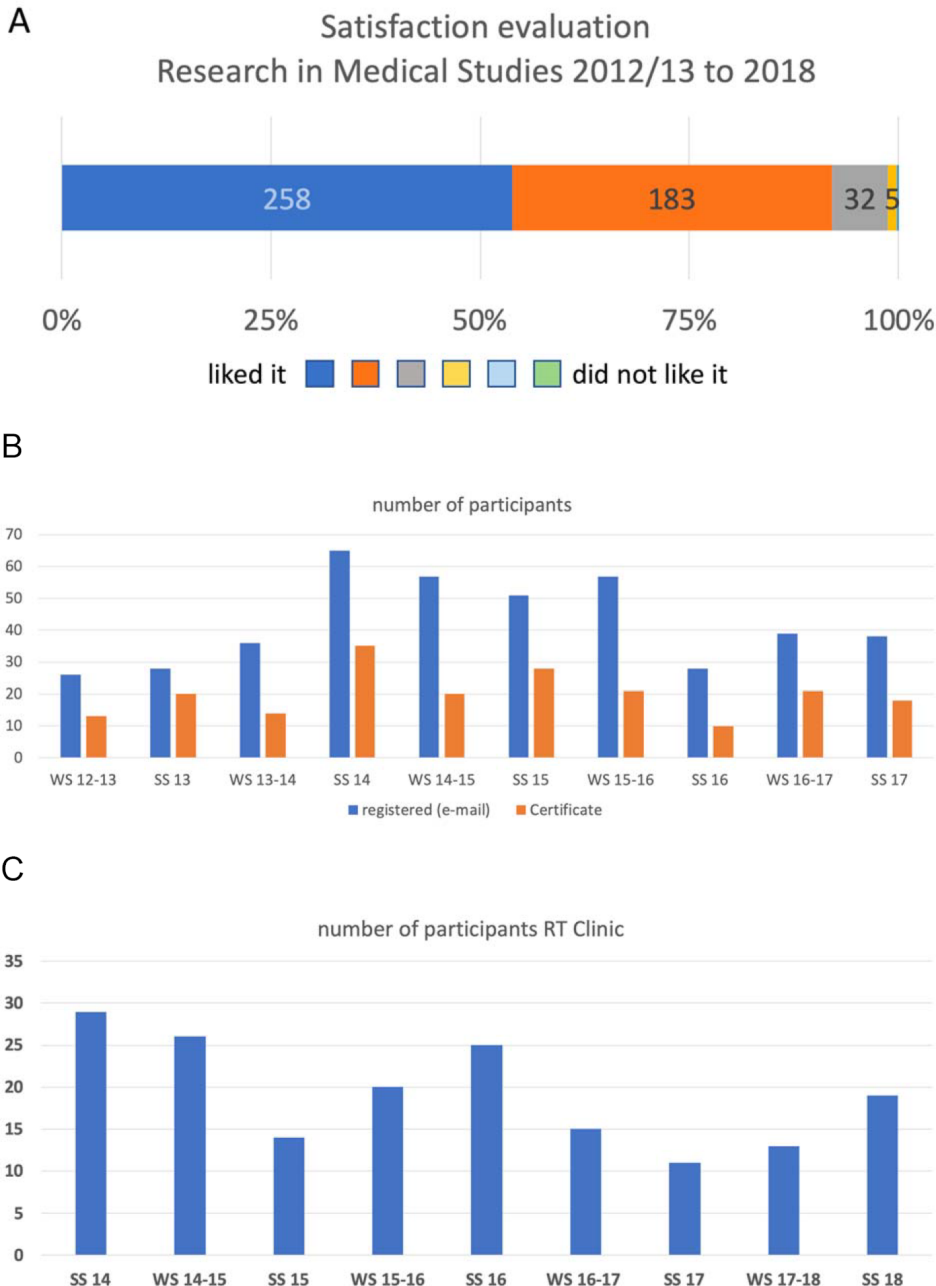
(A) Aggregated satisfaction evaluation of the event Research in Medical Studies on a Likert-type scale from "liked it" to “disliked it" in the period between winter semester 2012/13 and summer semester 2018 (B) Electronic registration for Research Track in the first study part and the percentage of participants who successfully completed Research Track in the period between WS 12/13 and SS 17. (C) The number of participants in Research Track of the second study part in the period between SS 14 and SS 18.

About 120-150 students of each first semester regularly participated in the voluntary informative events RiMS in the studied period, and about 40-70 students in the consecutive laboratory visits. The latter group of students judged the event as highly satisfactory (1.5±0.14 on a 6-level Likert scale from “liked it” (1) to “disliked it” (6); mean±SD; 12 semesters from WS 12/13 to SS 18 were considered, see figure 3 (Fig. 3)). In each semester, about 40% male (m) students and 60% female (f) students participated in the informative event RiMS (n=197 male and 303 female students; the evaluation was carried out by the participants who had attended the lecture and the laboratory visits; response rate about 80%).

#### Research Track (RT)

**Figure 4:**
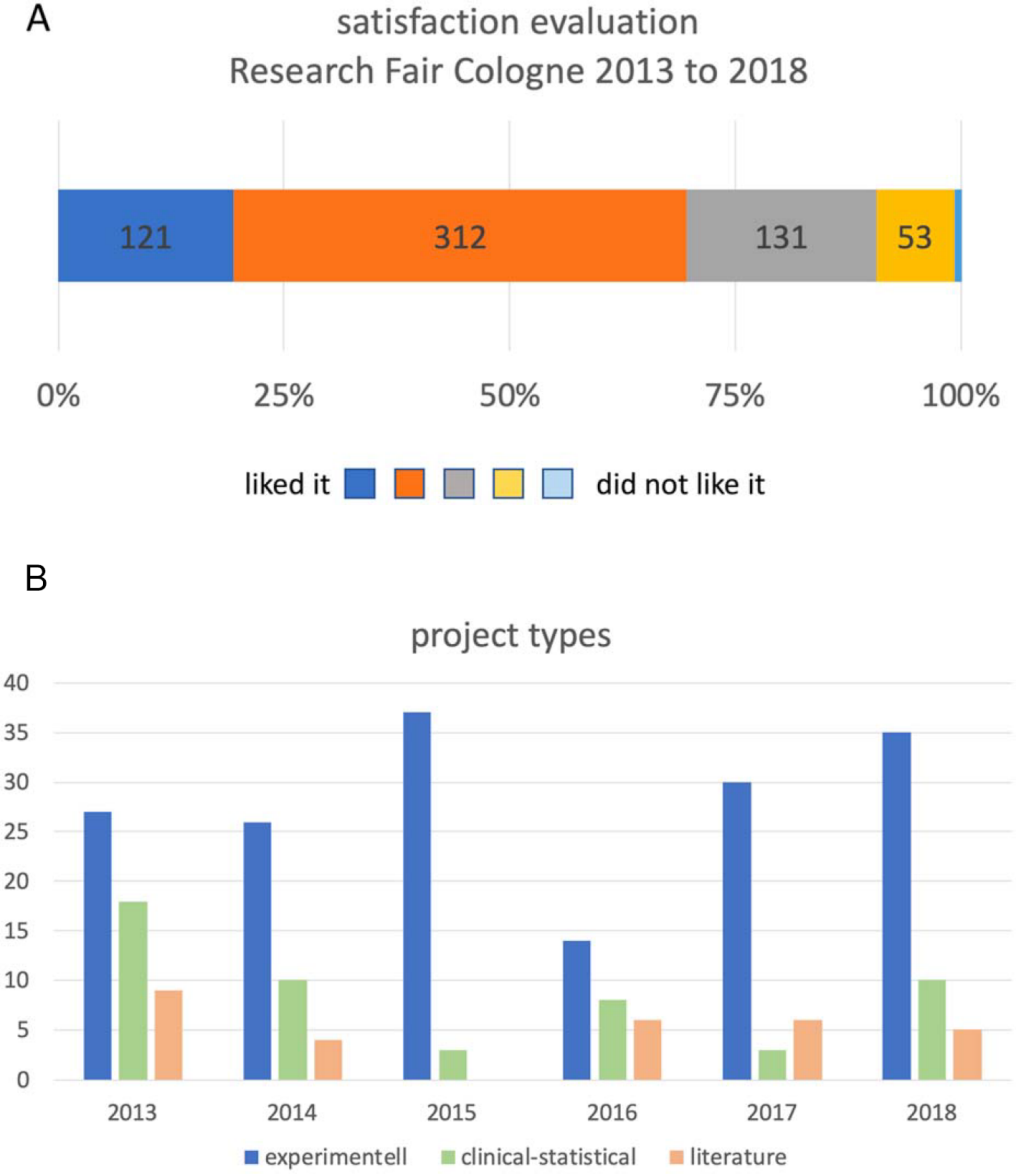
(A) Aggregated satisfaction evaluation of the event "Research Fair Cologne" on a Likert-type scale from "liked it" to "disliked it" in the period between 2013 and 2018 (B) Results of the outcome evaluation of the Research Fair. The absolute number of projects mediated and carried out at the event between 2013 and 2018 is shown. The projects are divided into experimental, clinical-statistical and literature projects.

Of the all the students in the second pre-clinical semester, an average of 21% of each cohort (approx. 40 persons) registered for participation in the program through e-mail; the number of successful participants averaged 47% of all those registered (this corresponds to approx. 10% of each cohort, survey period WS 2012/13-SS 17, see figure 4 (Fig. 4), point A). In the pre-clinical part of the research track, approximately 35% male students and 65% female students participated in each semester (evaluation data: 69 m, 129 f). The response rate of the evaluation, measured in relation to the number of registered participants of the respective semester, was 52% on average for both stages of study in the considered period. 

In the clinical part of the research track, an average of 20 participants registered per semester (corresponding to 12% of the total cohort of approximately 170 students, see figure 4 (Fig. 4), point B). 

Satisfaction with the pre-clinical Research Track was evaluated by the students as an average of 1.78±0.19 (mean±SD; pooled evaluation data from WS 12/13-WS 17/18) and in the clinical stage as an average of 1.73±0.14 (on a 6-level Likert scale from “liked it” (1) to “disliked it” (6); mean±SD; pooled evaluation data from SS 14-SS 18; evaluation sheet, see attachment 3 ). In the pre-clinical stage, English as the language of instruction was rated positively by the participants 1.7±0.4 (on a 6-level Likert scale from “liked it” (1) to “disliked it” (6); mean±SD, pooled evaluation data from WS 12/13-WS 17/18, see table 1 (Tab. 1)).

#### Research Fair

**Figure 5:**
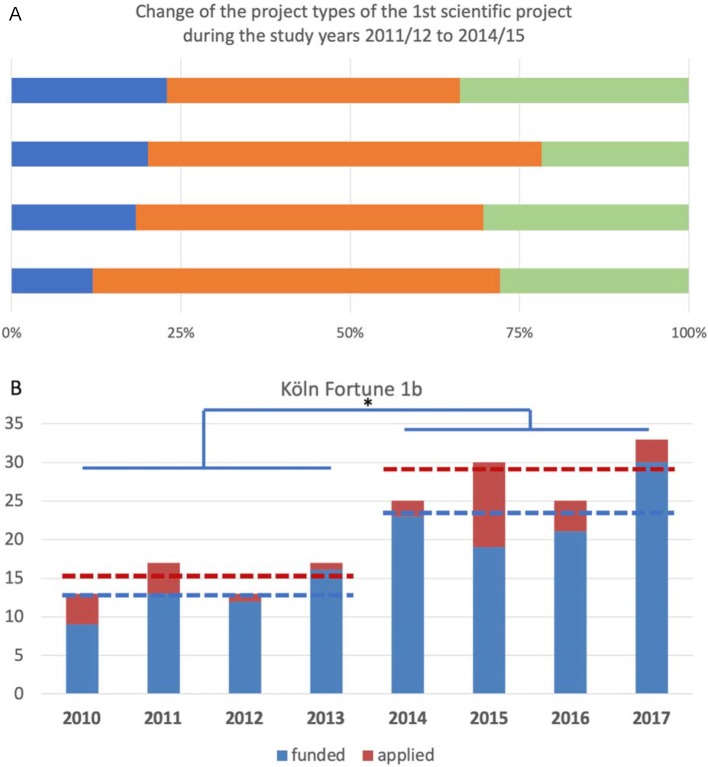
(A) Developmental distribution of the Scientific Project types in the first study stage based on the analysis of the project titles in the student files of the academic years 2011/12 to 2014/15. The projects are divided into experimental, clinical-statistical and literature projects. (B) Comparison of the number of Köln Fortune Projects applied for and accepted in the years 2010 to 2017. 15 projects were applied for on average (red dotted lines) in the years 2010-2013 and 27.7 projects in the years 2014-2017 (p=0.0012; two-sided t-test). On average (blue dotted lines), 12.5 projects were approved in 2010-2013 and 23.3 projects in 2014-2017 (p=0.0084; two-sided t-test).

Every year, an estimated 150 - 200 students and 100 scientists from an average of 30 different clinics and institutes take part in the Research Fair with approximately 50-70 posters. The evaluation of student satisfaction of the Research Fair resulted in a rating of 2.25±0.21 (on a 5-level Likert scale from “liked it” (1) to “disliked it” (6); mean±SD; pooled evaluation data from 2013-2018, see figure 5 (Fig. 5), point A). The students rated the item “I got a good overview of the faculty’s research” with 2.42±0.16 and the question “I got to know interesting Working Groups” with 2.37±0.18 (on a 5-level Likert scale from “agree” (1) to “disagree” (6); mean±SD; pooled evaluation data from 2015-2018). On average, approximately 48 of the students indicated that they potentially found a project (evaluation data from 2015-2018). The response rate can only be given approximately, since the Research Fair does not require participants to register or enroll. On average 105 (104.2±41.4; mean±SD; pooled evaluation data from 2013-2018) students evaluated the RFC, which corresponds to about two thirds of the participants. 

The outcome evaluation showed that on average 42 projects (41.8±8.4) were placed on the Research Fair. This corresponds to an average placement rate of approximately 1:4 to 1:5 per student participant. On average, 67.3% of these were experimental, 20.7% clinical-statistical and 11.9% literature work (see figure 5 (Fig. 5), point B). In 2018, 94 % of the projects offered stated that a publication was planned. The response rate to the outcome evaluation was 67.5%±11.7 on average.

Over time, an increase in the number of experimental projects can be shown (based on the titles of the performance records “Scientific Project”). The number of experimental projects as a proportion of all scientific projects increased from 8% in the academic year 2011/12 to 19% in the academic year 2014/15 (see figure 5 (Fig. 5), point A). At the same time, a significant increase in the submitted and successful Köln Fortune applications for student research projects can be observed from 2014 (one year after the first FBK) (see figure 5 (Fig. 5), point B). In the years 2010-2013 an average of 15±2.3 applications were submitted and in the years 2014-2017 an average of 27.7±3.8 applications were submitted (see figure 5 (Fig. 5), point B; t-test p=0.001). Of these, an average of 12.5±2.9 applications were approved in the same period before RaMS and an average of 23.3±4.7 applications were approved after its implementation (see figure 5 (Fig. 5), point B; t-test p=0.008).

#### Scientific Projects

With 189 students enrolled in the semester, a total of 411 pooled evaluation forms were submitted for Scientific Project I (pre-clinical stage) (response rate 54%).

The students rated the basic idea of conducting SPs as slightly positive with an average of 6.0 points (Likert scale 1-10, SD 2.5), better with regard to the project they had conducted themselves (average 7.0, SD 2.5). There is a moderate mathematical correlation between the ratings (r=0.53; Pearson). The students work in median 60 h (25%-quartile 32 h and 75%-quartile 120 h). Only 6 out of 411 (1.5%) evaluations reported that the students felt “exploited”.

## Discussion

With the introduction of the model curriculum in human medicine in Cologne, competence-oriented scientific education was integrated into the curriculum as early as 2003 with the implementation of the Scientific Projects [19]. In addition, introductory (RiMS), advanced (RT) and supporting courses (RFC) were implemented in the following years, which are continuously offered and attended by the lecturers and students of the Medical Faculty. This shows that it is principally possible to include student scientific work in the core curriculum of medical studies as a proof of achievement, which is required by the WR and also the MFT (in chapters 6 and 14a of the NKLM). Approximately 380 SP are completed per year in the pre-clinical stage and approximately 340 SP in the clinical stage.

It should not be forgotten that the Cologne model is merely a possible implementation of the requirements of WR and MFT, which is now more longitudinally structured at the Medical Faculty in Cologne under the label “Research and Medical Studies (RaMS)”. The model focuses on the support of top-level and broad-based scientific research. SP and RiMS ensure the basic qualification of all students, while RT and RFC particularly target the top group of students. Similarly complex, albeit differently structured programs (e.g. Sideres et al. 2018, [13]) also show a successful science-oriented career development of the participants. 

The acceptance of the individual optional curricular elements by the students is very positive. The Scientific Projects as a mandatory curricular component still perform positively overall (at least with regard to their own projects). With a median of 60 hours, the time spent on the projects is on average within the time frame provided for this in the study regulations (60-120 hours), although it is at the lower end of the scale. This fits in with the students' statements that only very few students (1.5%) report to have been exploited.

Since the competencies required by the NKLM (Chapter 14a, Sections 2 and 3 [http://www.nklm.de]) are already taught in the SP, students who attend the voluntary preparative and advanced courses (RiMS, RT, RFC) have the opportunity to knowingly plan their careers in a scientifically guided manner (or to promote young scientists in the sense of the faculty). Only some of the students take advantage of the voluntary elements. The number of participants who complete the Research Track amounts to approx. 10% of the students in a year and is thus comparable to similar programs (MFT Workshop for Teaching Scientific Competence in Medical Studies (Würzburg 2017)). In our opinion, the performance of experimental work represents the entire research process from the formulation of the research question to the conduction of experiments and the evaluation and interpretation of the data. Literature work in the context of Scientific Projects (which are not systematic reviews) does not fulfill this. In this context, the increase in experimental work seems to represent a qualitative improvement in terms of mapping the entire research process. Whether the demonstrated effects on the scientific activities (increase in experimental projects and increased application or success with application-related research grants) are causally related to the RaMS program or not, cannot be proven. However, the close temporal relationship to the newly introduced teaching elements and effects studied suggests that this is the case.

At the same time, the placement rate in the context of the RFC of approx. 25% of the participating students shows a pleasingly high efficiency of this measure for the students. Furthermore, these and other elements of the RaMS program obviously also generate direct and lasting added value for the teachers: The consistently high level of participation by the faculty in the RFC, RT and also the SP indicates this as well.

In addition to the structural integration of the program in the examination regulations of the model study program, it should be emphasized that such a comprehensive program can only be maintained with the support of a staff member – in Cologne in the form of a research associate with two student assistants (cf. [20]). In addition to the coordinating tasks (particularly the recruitment of lecturers in RT, but also in contact with the Medical Faculty as the governing body of the RFC), the strategic development of the program is the main focus.

Although or perhaps because of the positive experience for the past 15 years with the model curriculum at the Medical Faculty in Cologne in conveying science in medical studies in accordance with the recommendations of the WR and MFT, the next consecutive steps in the further development of the concept are already becoming apparent:

- The recently founded Graduate School of Human and Dental Medicine (GSHZ Cologne) guides the qualified paracurricular science program in the first own scientific achievement, the doctorate.
- With the faculty's decision to change the academic regulations for the Model Study Program 2017, the Cologne Science Skills Lab (KWiS^med^) was founded, whose task is the methodical hands-on preparation of students for training in scientific projects (and doctorates).
- The reorganization of teaching in the first study stage, which is expected to start in 2021, will be challenging. This will also involve an even better combination of traditional and new elements in the scientifically-oriented curriculum of the model study program.

## Conclusion

Taken together, it becomes clear, particularly through the description of the complex intervention to improve the teaching of science in the study program, that a simple strategy, for example one oriented towards research-based learning [21], cannot be effective without curricular embedding and guidance [13], [22]. Even if the overall effects of the programs can be shown, proof of their effectiveness in the sense of “clarification studies” [23] must still be provided in future individual cases. 

## Competing interests

The authors declare that they have no competing interests. 

## Supplementary Material

Evaluation Research in Medical Studies

Evaluation Research Track

Evaluation Research Fair

## Figures and Tables

**Table 1:**
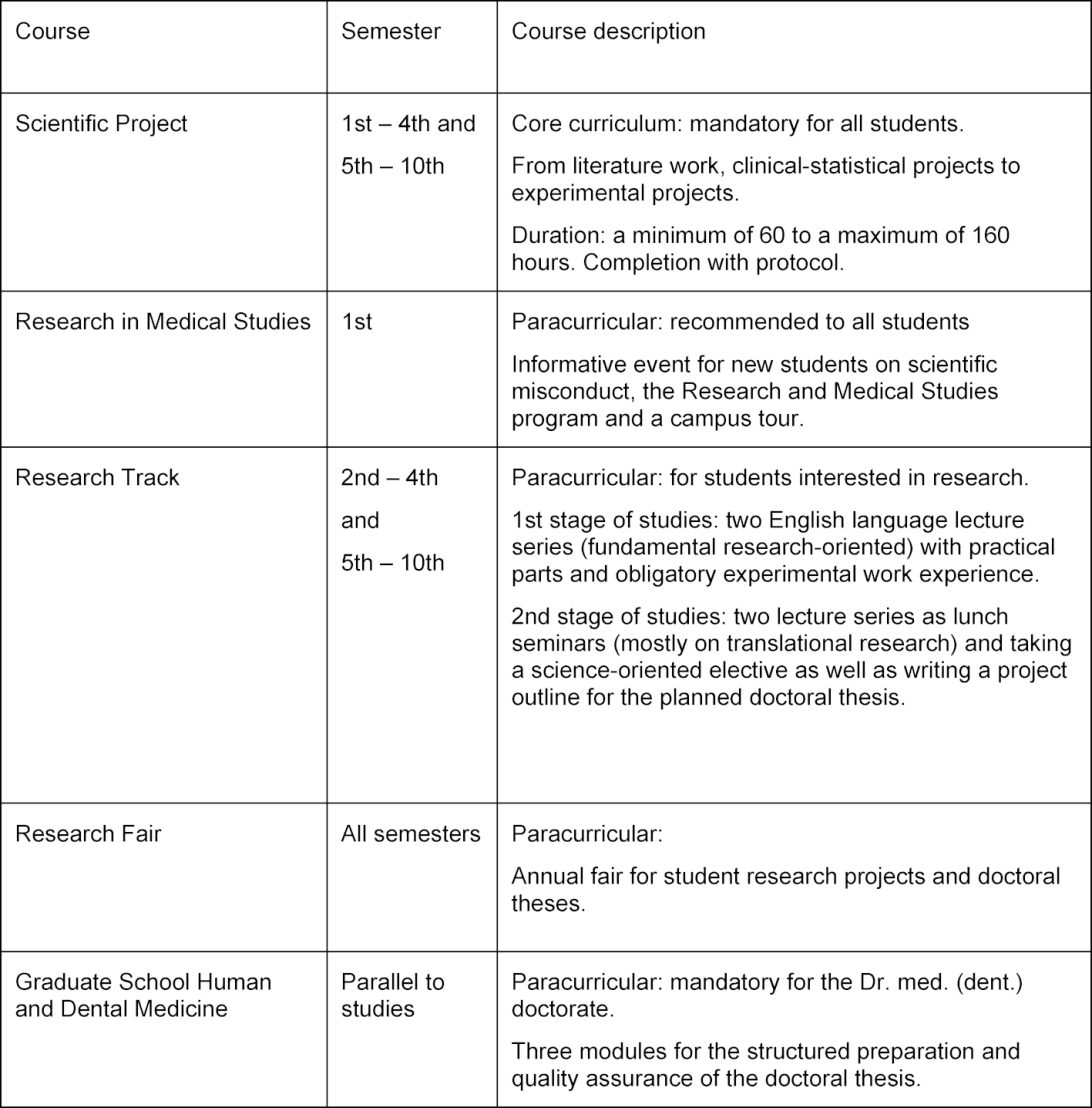
Overview of the program Research and Medical Studies (RaMS)

## References

[1] Wissenschaftrat (2018). Neustrukturierung des Medizinstudiums und der Änderung der Approbationsordnung für Ärzte - Empfehlungen der Expertenkommission zum Masterplan 2020; DRS 7271 -18.

[2] Ratte A, Drees S, Schmidt-Ott T (2018). The importance of scientific competencies in German medical curricula - the student perspective. BMC Med Educ.

[3] Nationale Akademie der Wissenschaften Leopoldina, Medizinischer Fakultätentag (2019). Die Bedeutung von Wissenschaftlichkeit für das Medizinstudium und die Promotion.

[4] World Federation for Medical Education (2015). Basic Medical Education. WFME Global Standards for Quality Improvement. The 2015 Revision.

[5] Epstein N, Huber J, Gartmeier M, Berberat PO, Reimer M, Fischer MR (2018). Investigation on the acquisition of scientific competences during medical studies and the medical doctoral thesis. GMS J Med Educ.

[6] Flexner A (1910). Medical education in the United States and Canada: a report to the Carnegie Foundation for the Advancement of Teaching.

[7] Frank JR, Snell L, Sherbino J (2015). CanMEDS 2015 Physician Competency Framework.

[8] Hautz SC, Hautz WE, Keller N, Feufel MA, Spies C (2015). The scholar role in the National Competence Based Catalogues of Learning Objectives for Undergraduate Medical Education (NKLM) compared to other international frameworks. GMS Ger Med Sci.

[9] Hautz SC, Hautz WE, Feufel MA, Spies CD (2016). What makes a doctor a scholar: a systematic review and content analysis of outcome frameworks. BMC Med Educ.

[10] Von Wichert P (2014). Medizinische Ausbildung in Zeiten der Bologna-Reform: Wesensfremde Kleinteiligkeit. Dtsch Arztebl.

[11] Wissenschaftsrat (2014). Empfehlungen zur Weiterentwicklung des Medizinstudiums in Deutschland auf Grundlage einer Bestandsaufnahme der humanmedizinischen Modellstudiengänge Drs.

[12] Stern DT, Papadakis M (2006). The developing physician--becoming a professional. N Engl J Med.

[13] Sideris M, Hanrahan J, Staikoglou N, Pantelidis P, Pidgeon C, Psychalakis N, Andersen N, Pittaras T, Athanasiou T, Tsoulfas G, Papalois A (2018). Optimizing engagement of undergraduate students in medical education research: The eMERG training network. Ann Med Surg (Lond).

[14] Fang D, Meyer RE (2003). Effect of two Howard Hughes Medical Institute research training programs for medical students on the likelihood of pursuing research careers. Acad Med.

[15] Ommering BWC, van den Elsen PJ, van der Zee J, Jost CR, Dekker FW (2018). Using an Extracurricular Honors Program to Engage Future Physicians Into Scientific Research in Early Stages of Medical Training. Med Sci Educ.

[16] Cluver J, Book S, Brady K, Back S, Thornley N (2014). Engaging medical students in research: reaching out to the next generation of physician-scientists. Acad Psychiatry.

[17] Steffen J, Grabbert M, Pander T, Gradel M, Köhler LM, Fischer MR, von der Borch P, Dimitriadis K (2015). Finding the right doctoral thesis - an innovative research fair for medical students. GMS Z Med Ausbild.

[18] Stosch C, Lehmann K, Herzig S (2008). Time for Change - Die Implementierung des Modellstudiengangs Humanmmedizin in Köln. ZFHE.

[19] Zims H, Karay Y, Neugebauer P, Herzig S, Stosch C (2019). Fifteen years of the cologne medical model study course: has the expectation of increasing student interest in general practice specialization been fulfilled?. GMS J Med Educ.

[20] Eckel J, Schüttpelz-Brauns K, Miethke T, Rolletschek A, Fritz HM (2017). The inventory as a core element in the further development of the science curriculum in the Mannheim Reformed Curriculum of Medicine. GMS J Med Educ.

[21] Huber L, Huber L, Hellmer J, Schneider F (2009). Warum Forschendes Lernen no¨tig und möglich ist. Forschendes Lernen im Studium.

[22] Mayer RE (2004). Should there be a three-strikes rule against pure discovery learning? The case for guided methods of instruction. Am Psychol.

[23] Cook DA, Bordage G, Schmidt HG (2008). Description, justification and clarification: a framework for classifying the purposes of research in medical education. Med Educ.

