# Supplementary material for: Studies on acceptance, evaluation and impact of the Cologne program “Research and Medical Studies”
Source: GMS J Med Educ. 2020 Feb 17;37(1):Doc5. doi: 10.3205/zma001298 (PMC7105762; doi:10.3205/zma001298)
Supplement: Evaluation Research in Medical Studies [file JME-37-5-s-001.pdf]

Appendix 1

# Evaluation Research in Medical Studies

Gender ☐ M ☐ F

**Motivation:** (Multiple answers possible)

| | |
|---|---|
| Scientific interest | ☐ |
| Scientific Project | ☐ |
| Curiosity | ☐ |
| Other | |

Please evaluate the individual parts of the event:

| | Liked it … Disliked it |
|---|---|
| Research Physician | ☐☐☐☐☐☐ |
| Introduction | ☐☐☐☐☐☐ |
| Science bvmd | ☐☐☐☐☐☐ |
| Research Track | ☐☐☐☐☐☐ |
| Scientific Projects/Dissertation | ☐☐☐☐☐☐ |
| Lab visits | ☐☐☐☐☐☐ |

**Overall evaluation of the event:**

Liked it ☐☐☐☐☐☐ Disliked it

**Criticism, suggestions, compliments:**
