# Supplementary material for: Studies on acceptance, evaluation and impact of the Cologne program “Research and Medical Studies”
Source: GMS J Med Educ. 2020 Feb 17;37(1):Doc5. doi: 10.3205/zma001298 (PMC7105762; doi:10.3205/zma001298)
Supplement: Evaluation Research Track [file JME-37-5-s-002.pdf]

Appendix 2

Evaluation Research Track

Gender ☐ M ☐ F Semester ☐

Motivation: (Multiple answers possible)

	Scientific interest	☐
	Scientific Project	☐
	Curiosity	☐
	Other	

	Liked it					Disliked it
16.1. Dr. N. N.	☐	☐	☐	☐	☐	☐

Overall evaluation of the lecture series:

	Suitable					Unsuitable
Time	☐	☐	☐	☐	☐	☐
Day	☐	☐	☐	☐	☐	☐

	Agree					Disagree
English Language	☐	☐	☐	☐	☐	☐

	Liked it					Disliked it
Overall impression	☐	☐	☐	☐	☐	☐

Homepage Research Track:

Visited ☐ Yes ☐ Nein

	Liked them					Disliked them
Methods	☐	☐	☐	☐	☐	☐

Criticism, suggestions, compliments:
