# Supplementary material for: Studies on acceptance, evaluation and impact of the Cologne program “Research and Medical Studies”
Source: GMS J Med Educ. 2020 Feb 17;37(1):Doc5. doi: 10.3205/zma001298 (PMC7105762; doi:10.3205/zma001298)
Supplement: Evaluation Research Fair [file JME-37-5-s-003.pdf]

Appendix 3

Evaluation Research Fair

Semester: ___ ← please enter your semester here

1 = agree/liked | 5 = disagree/disliked

		1	2	3	4	5
1	I got a good overview of the faculty's research	O	O	O	O	O
2	I got to know interesting Working Groups	O	O	O	O	O
3	I'm visiting the Research Fair for the following reason<br>Searching for: Scientific Project Preclinic<br>Scientific Project Clinic<br>Dissertation<br><br>Other:	O<br>O<br>O				
4	I found a potential:<br>Scientific Project Preclinic<br>Scientific Project Clinic<br>Dissertation<br><br>I didn't find a potential project.	O<br>O<br>O<br><br>O				
6	Overall evaluation of the Research Fair (1= best mark / 5= worst)	O	O	O	O	O

Your opinion is important to us! Please write down your criticism, suggestions or compliments in the text field below:
